# Spontane Infrastrukturen: Der Jeep und die Erschaffung postkolonialer Räume in der Nachkriegszeit, 1940er bis 1960er Jahre

**DOI:** 10.1007/s00048-025-00410-y

**Published:** 2025-03-05

**Authors:** Heinrich Hartmann

**Affiliations:** Wissensgeschichte moderner Gesellschaften, Fakultät für Geistes- und Sozialwissenschaften, Helmut-Schmidt-Universität Hamburg, Hamburg, Deutschland

**Keywords:** Jeep, Zweite koloniale Eroberung, U.S. Army, Brooks Stevens, Henry Kaiser, Willys Overland, Jeep, Second colonial occupation, U.S. Army, Brooks Stevens, Henry Kaiser, Willys Overland

## Abstract

Der Jeep gehörte mit zu den wichtigsten Ausrüstungsgegenständen der US-amerikanischen Armee im Zweiten Weltkrieg und wurde entsprechend im und nach dem Krieg heroisiert. Gleichzeitig war die Nachkriegsverwendung von hunderttausenden von Jeeps nicht geklärt. Die Umnutzung als landwirtschaftliches Gerät erwies sich als schwierig. Stattdessen wurden die Jeeps häufig in verschiedenen geografischen Kontexten in neue Nutzungen überführt und stellten gerade in schlecht erschlossenen Teilen der Welt eine neue Form von spät- und postkolonialer Infrastruktur dar. Dabei waren Jeeps weit mehr als einfache Transportgeräte. Sie ermöglichten den Zugang zu abgelegenen Regionen und Dörfern – mit dem Anspruch, diese auch an einer scheinbar globalen Informationsgesellschaft teilhaben zu lassen.

Gleichzeitig begann der Aufstieg des Jeeps als Statussymbol der gehobenen US-amerikanischen Mittelschicht. Dabei spielt neben der Technik auch das Design eine entscheidende Rolle, um den Jeep mit den sich verändernden Raumentwürfen der Nachkriegszeit zusammenzudenken. Insbesondere Brooks Stevens und Henry Kaiser hatten einen entscheidenden Anteil daran, das frühere Militärfahrzeug einem ganz anderen Nutzen zuzuführen, der aber in vielerlei Hinsicht weiterhin Teil der *modernizing mission* der Nachkriegszeit war. Dieser Aufsatz versteht diese beiden Verwendungen von Jeeps nicht als akzidentelle Parallelentwicklungen, sondern versucht sie als zwei Seiten der gleichen Medaille zu lesen.

## Abstract (en)

The jeep was one of the most important pieces of equipment used by the US American army in the Second World War and was heroized accordingly during and after the war. At the same time, the post-war use of hundreds of thousands of jeeps was unclear. Repurposing them as agricultural equipment proved to be difficult. Instead, Jeeps were often put to new uses in different geographical contexts and represented a form of late and post-colonial infrastructure, especially in parts of the world with little access to transportation and road networks. However, jeeps were far more than simple transportation devices. They enabled access to remote regions and villages, with the aim of allowing them to participate in a seemingly global information society.

At the same time, the Jeep began its rise as a status symbol of the upper American middle class. Design played a decisive role in bringing the Jeep together with the changing mental maps of the post-war period. Brooks Stevens and Henry Kaiser in particular played an important role in giving the former military vehicle a completely different use, although in many respects it remained part of the modernizing mission of the Postwar period. This essay understands these two uses of the Jeep not as coincidental parallel developments, but reads them as two sides of the same coin.

Die direkte Nachkriegszeit war geprägt vom Jeep. Im Zuge der alliierten Befreiung Deutschlands von den Nazis wurde dieses Fahrzeug zum Signum politischen und gesellschaftlichen Wandels: Die GIs, die der besiegten Bevölkerung in ihren Jeeps begegneten – durch die offene Chassiskonstruktion nahbarer, durch den technisch überlegenen Motor gleichzeitig unerreichbarer als die deutschen Soldaten – waren ein Bild, das die Nachkriegszeit und viele persönliche Erinnerungen daran prägte (Iken 2018).

Doch die starke symbolische Kraft, die sich mit dem Jeep verband, beschränkte sich bei Weitem nicht nur auf die US-amerikanische Besatzungszone in Deutschland. Vielmehr rollte der Jeep schnell in sehr verschiedenen Missionen durch die Welt. Er durchquerte Wüsten, brachte Ärzt:innen in entlegene Regionen und tauchte oft unangekündigt in Dörfern auf, wo sein „power outlet“ etwa die mobile Installation von Kinovorführungen ermöglichte (Kim 2016: 70). Nachrichten, Aufklärungsfilme, aber auch Hollywoodklassiker kamen mit den Jeeps in die Dörfer des „globalen Südens“ und verstärkten die Illusion eines mittels Kommunikation vernetzten *global village*.

Die Tatsache, dass das Getriebe des Jeeps mit seiner hohen Übersetzung – gestützt von einem neuartigen und effizienten Kühlsystem und dem ersten massenhaft verbauten Allradantrieb – fast überallhin vordringen konnte, auch in Regionen, die infrastrukturell kaum erschlossen schienen, machte ihn selbst zu einer Infrastruktur, die sich auf vielfältige Weise mit den Versprechungen sozioökonomischer Modernisierung verband (van Laak 2020). Er ermöglichte in den Augen von Militärs, aber auch späterer Nutzer:innengruppen die ständige spontane Erreichbarkeit abgelegener Gebiete. Aus Sicht dieser Nutzer:innen waren abgelegene Gebiete nicht durch Straßen- oder Schienennetze miteinander verbunden, sondern durch das Vorhandensein einer ausreichenden Menge von Jeeps.

War die Spontaneität automobiler Erreichbarkeit zu Beginn des Autozeitalters in den ersten Jahrzehnten des 20. Jahrhunderts fester Bestandteil der neuen Mobilität – von den frühen Automobilmodellen einschliesslich des Ford T in den USA erwarteten die Entwickler zunächst, dass sie unabhängig von Straßen und festen Verkehrswegen durch die Landschaft fahren sollten –, so war dies mit dem Ausbau des für Autos vorgesehenen Straßennetzes ab den 1920er Jahren zunehmend vorbei (Volti 2004: 46; Möser 2002: 89–95; Kline & Pinch 1996). Der Ausbau stationärer Infrastruktur mit der Fixierung automobiler Technik auf diese Straßen schränkte die „Überlandfähigkeit“ mobiler Systeme ein und trug damit auch zur Wahrnehmung von Abgelegenheit, ja Abgeschiedenheit von Gebieten abseits des Straßennetzes bei. Diese zu überbrücken wurde zur Aufgabe von Spezialfahrzeugen. Technisches Gerät stand damit in Analogie zu den neu ausgebauten Verkehrswegen und sicherte auf spontane Weise den Zugang zu diesen Räumen begrenzter Erreichbarkeit ab.

Dieser Aufsatz folgt der Hypothese, dass der Jeep – beziehungsweise die Aushandlung des ihm zugrunde liegenden technologischen Komplexes in der frühen Nachkriegszeit – eine Lücke füllte, die das Fahrzeug zu mehr als nur einem individuellen Fortbewegungsmittel machte. Es stellte die prinzipielle Erreichbarkeit abgelegener Regionen zu praktisch jedem Zeitpunkt sicher. Verbunden damit war auch die Verknüpfung mit der Frage eines bestimmten postkolonialen Machtdispositivs: War es in der Phase des Kolonialismus die militärische Erreichbarkeit durch entsprechende Kampagnen, so schien nun die Verbindung mit Entwicklungsprogrammen und der Anschluss an Informationssysteme diese Rolle zu erfüllen. Eine solche Rolle als spontane Infrastruktur verband die verschiedenen räumlichen Neuentwürfe der frühen Nachkriegszeit miteinander.

Wenn man das in den letzten Jahren diskutierte Konzept von Infrastrukturen mit den inzwischen klassischen Perspektiven auf die soziale Konstruktion von Technik zusammendenkt, wie mir dies im Fall des Jeeps als logisch erscheint, dann geht dies nicht ohne einen Blick auf die räumliche Komponente der technischen Infrastruktur zu werfen. Ein Spezifikum der Infrastruktur als „kollektivem Unterbewussten“ (van Laak & Burchardt 2023: 1–18; Hack 2023: 133–150) ist, dass sie Räume mitgestaltet und die Akzeptanz und Manipulation von Technik damit gleichzeitig Pfade der Raumaneignung und Ästhetisierung vermittelt (ERC-Projekt: Off the Road; Mom 2015: 490–498; Kline 2000). Infrastruktur in einem solchen postkolonialen Sinne trägt damit einerseits dazu bei, soziale und ökonomische Machtstrukturen zu festigen oder immerhin zu lenken. Andererseits kreiert sie diese sozialen Zusammenhänge auch mit – in diesem Fall durch die Durchdringung von Räumen. Sie wandelt den Blick auf die vermeintlich harten Brüche politischer und sozioökonomischer Veränderungen in der Phase der Dekolonisierung.

Auf methodischer Ebene ist ein solcher Infrastrukturbegriff offen gegenüber Momenten der Akzeptanz und Aushandlung. Die hier angedachte spontane Infrastruktur hat eine Realität jenseits der Pläne derjenigen, die sie ursprünglich geplant haben. Bewusst möchte ich dabei die Frage sozialer Konstruktion der Jeep-Technologien (Pinch & Bijker 1987) in einer postkolonialen Perspektive diskutieren und hierdurch verschiedene Raumgestaltungen miteinander in Verbindung setzen. Der Wechsel der Nutzer:innengruppen, der sich im Übergang von militärischer zu ziviler Nutzung in den frühen Nachkriegsjahren vollzog, fand in unterschiedlichen Weltregionen statt: in den sich entkolonisierenden Ländern der ehemaligen europäischen Kolonialreiche, aber auch an der neu entstehenden „suburban frontier“ in den USA (Sklare & Greenblum 1979: 287–291). Nicht zuletzt ist dies gerade durch die aktive Rolle von Unternehmer:innen und industriellen Designer:innen zu erklären, die die notwendigen Übersetzungen und Anpassungen vollzogen und damit eine solche Verbindung überhaupt erst ermöglichten.

Im Folgenden werde ich zunächst knapp auf die Entstehungsgeschichte des Jeeps im Kontext des Zweiten Weltkriegs eingehen. Gerade zum Kriegsende wurde der Jeep zu einem technischen Gegenstand, dessen Rolle außerhalb des Kriegseinsatzes zu klären war. Diese Aushandlungsprozesse werden in einem zweiten Abschnitt im Vordergrund stehen, der zeitlich auf die frühen Nachkriegsjahre fokussiert. Ein dritter Abschnitt wird sich mit der Neuentdeckung relevanter Nutzer:innengruppen im gänzlich anders gearteten sozialen Kontext der Dekolonisierung und der US-amerikanischen Suburbia in den 1940er bis 60er Jahren beschäftigen, bevor im letzten Schritt versucht werden soll, Querbeziehungen zwischen diesen Nutzungsformen zu identifizieren.

## Vom Weltkriegshelden …

Die Rolle als Schlüsseltechnologie einer spät- und postkolonialen Modernisierung war dem Jeep nicht in die Wiege gelegt oder gehörte gar, technikhistorisch gesprochen, zu dessen Skript oder dem Szenario der vorhergesehenen Nutzung (Akrich 1992: 208). Motor, Kühlung und *power outlet* waren für einen militärischen Zweck vorgesehen. Der Innovationsprozess, der dem Jeep zugrunde lag, verrät wenig über seine spätere Verwendung. Wohl aber verweist der militärische Ursprungskontext auf die sich wandelnden Nutzer:innengruppen, auf divergierende Interessen und die gestalterische Rolle, welche die Produzent:innen und Designer:innen des Jeeps schon in der Anfangsphase eingenommen haben, ganz im Sinne einer gegenseitigen Abhängigkeit von technischem und sozialem Wandel (Bijker 1995; Sørensen 2012: 123–144).

Mit dem Ende des Zweiten Weltkriegs war der Jeep wohl der wichtigste nichtpersonelle Kriegsheld und gleichzeitig das dritte Rad am Wagen der US-Armee. Dies resultierte aus einer Erfolgsgeschichte, die lange vor dem Kriegseintritt der USA begonnen hatte. Im Ersten Weltkrieg war die Bedeutung einer neuen (Auto‑)Mobilität zum ersten Mal in einem militärischen Kontext zum Tragen gekommen. Der massive Einsatz von LKWs für den Truppentransport hatte sich für die Alliierten als Vorteil erwiesen, die zurückhaltende Verwendung durch die deutsche Armee hingegen als strategischer Nachteil. Dies trug entscheidend zum Erfolg des LKWs ab den 1920er Jahren bei, der durch den Krieg berühmt geworden war (Möser 2002: 106–127). Dies galt ebenso für die nationalen Armeen wie auch für den zivilen Bereich. Gekoppelt war dieser Erfolg des automobilen Schwertransports allerdings an den Ausbau der Infrastruktur, denn die großen Fahrzeuge konnten nur auf Straßen mit der nötigen Kapazität und Leistungsfähigkeit fahren.

Aus militärischer Perspektive ergab sich hieraus eine Leerstelle bei der Beherrschung des Raums jenseits dieser ausgebauten Infrastruktur, denn hierfür kamen nur kleinere, leichte Fahrzeuge infrage. Das Quartermaster Corps der U.S. Army hatte bereits in den 1920er Jahren diesen Bedarf für neue leichte Aufklärungsfahrzeuge festgestellt und auch schon in den 1930er Jahren mit dem Gebrauch einiger Modelle experimentiert (Hyde 2013: 148; Jeudy & Tararine 1981: 19ff.). Doch den ersten Projekten war kein Erfolg beschieden, denn die meist nur leicht umgebauten zivilen Fahrzeugtypen (darunter ursprünglich auch der Ford T) erwiesen sich in schwerem Gelände als unbrauchbar (Seelinger 2006: 9). Angesichts der steigenden Wahrscheinlichkeit, einen Krieg auch außerhalb der eigenen geografischen und klimatischen Grenzen, weit entfernt von der infrastrukturell erschlossenen Situation in den USA selbst, führen zu müssen, wuchs um 1940 der Druck, eine neue tragfähige Lösung für die Bereiche Aufklärung und Transport der Truppen zu finden. Am 27. Mai 1940 wandte sich der Quartermastercorps mit einer Ausschreibung für ein „leichtes Aufklärungsfahrzeug“, das nicht mehr als eine Tonne wiegen und sich verschiedenen Geländesituationen anpassen können sollte, an 135 Autoproduzenten in den USA (ebd.). Die Vorgaben – insbesondere hinsichtlich des Gewichts – waren allerdings so rigide, dass sich kaum eines der Unternehmen dazu bereitfand, der Armeeführung ein entsprechendes Angebot senden zu können. Als erstes und lange Zeit einziges Unternehmen hatte Bantam im September desselben Jahres der Armeeführung einen Prototypen des Bantam Reconnaissance Car (BRC) zur Verfügung gestellt, kurz vor Ende der offiziellen Deadline. Um den Rücklauf auf die Ausschreibung zu verbessern, gab die Armeeführung Bantams Konkurrenten Willys und Ford eigenmächtig Zugang zu Bantams Modellen und Bauplänen, was es den beiden Konkurrenten doch noch ermöglichte, im November 1940 eigene Modelle (Willys Quad und Fords Pygmy) nachzureichen. Es verwundert vor diesem Hintergrund wenig, dass sich die drei Prototypen äußerlich recht ähnlich waren.

**Abb. 1:**
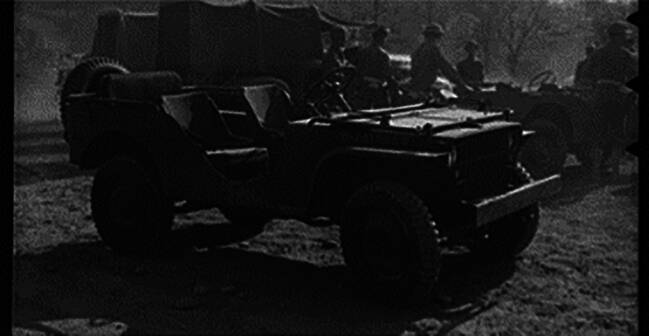
Das Bentham Reconnaissance Car in Fort Myers, 1941 (Cord McKenna, „The Bantam Reconnaissance Car“, *Butler County Historical*, https://www.butlerhistorical.org/items/show/47)

**Abb. 2:**
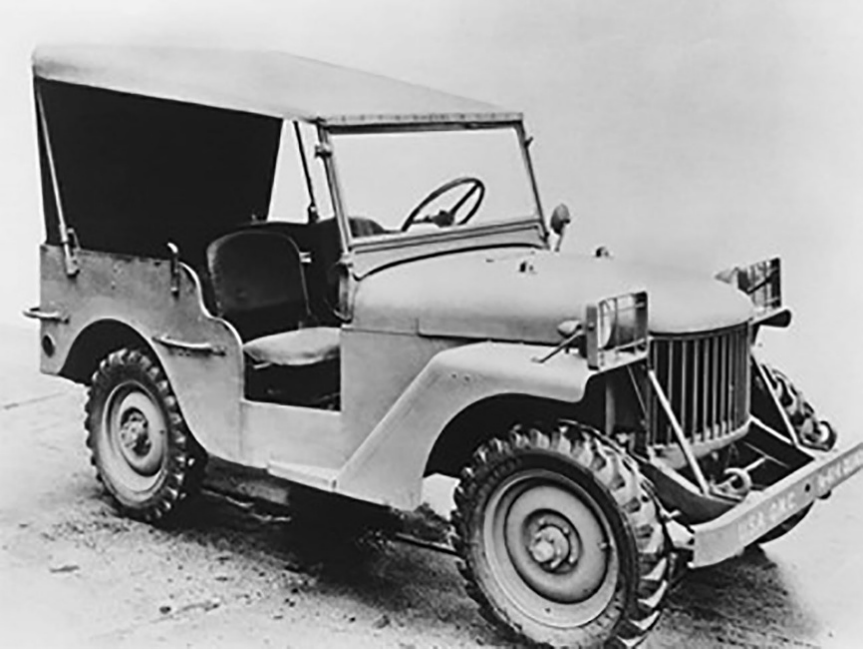
Willys Quad, 1940 (https://www.jeep.com/history/1940s.html)

**Abb. 3:**
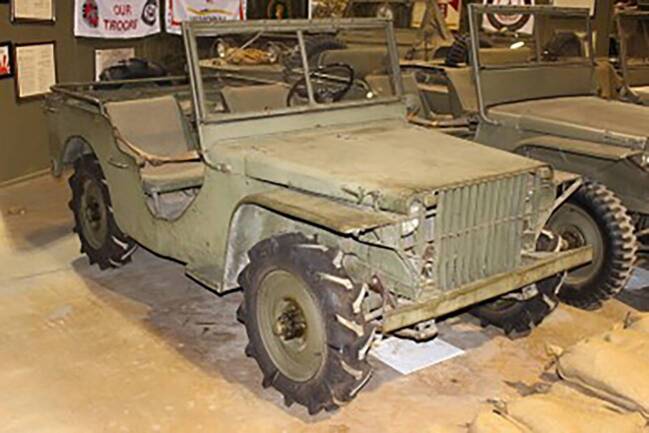
Ford Pygmy (https://de.wikipedia.org/wiki/Datei:Ford_Pygmy_jeep_pilot_vehicle.JPG)

Die Unterschiede lagen weniger im Chassisbau, auch wenn Willys es geschafft hatte, im Vergleich zu Bantam einige Zentimeter tiefer zu bauen und somit die Stabilität des Fahrzeugs in unwegsamem Gelände entscheidend zu verbessern (Hyde 2013: 148f.). Die eigentliche Neuerung des nachgelieferten Prototyps von Willys lag in der Kühlung und dem Motor mit hoher Übersetzung – dem sogenannten „*go devil“-*Motor, der mit 62 PS stärker war als die der Konkurrenten Bantam (45 PS) und Ford (40 PS). Der Motor hatte ein hohes Drehmoment, wurde jedoch durch das neuartige Kühlsystem vor Überhitzung geschützt. Es erlaubte, auch über längere Strecken in niedrigeren Gängen zu fahren. Die Kombination aus Kühlung und Motor resultierte in einer höheren Leistungsstärke des Quads in unwegsamem Gelände wie auch der Möglichkeit, den Motor als Traktionsmaschine oder als Stromgenerator zu nutzen.

Alle bei der U.S. Army eingereichten Modelle wogen ausnahmslos wesentlich mehr als die Vorgaben vorgesehen hatten. Für den Quad und sein späteres Nachfolgemodell, den MA, lag der Grund genau in Motor und Übersetzung, die viel schwerer waren als zunächst geplant. Wegen des bestehenden Zeitdrucks im Angesicht eines sich global ausweitenden Krieges entschied sich die Armee allerdings dafür, auf eine Neuausschreibung zu verzichten und stattdessen das Kriterium Gewicht flexibel zu handhaben: Sie bestellte bei allen drei Firmen zunächst jeweils 1.500 Exemplare der Fahrzeuge, die als „Jeeps“ in den allgemeinen Sprachgebrauch eingingen.^1^ Im Sinne der Rationalisierung der Produktion entschied sich die Armee im Juli 1941 für das Modell MA, das Serienmodell von Willys Quad, und gegen die Angebote von Bantam und Ford. Diese Vereinheitlichung ging damit einher, dass Willys’ Modell ab November 1941 auch in Lizenz von Ford mithergestellt wurde, womit die Massenproduktion auch für die Kriegszeit endgültig gesichert war (Hyde 2013: 148–152).

Bis zum Ende des Zweiten Weltkriegs produzierten Willys und Ford somit 647.343 Jeeps, inklusive der zusätzlich entwickelten Amphibienfahrzeuge, die an allen Schauplätzen des Krieges zum Einsatz kamen (ebd.: 152). Die ursprünglich vollkommen unspezifische Bezeichnung „Jeep“, die nicht nur die verschiedenen Prototypen umfasste, sondern sich auch immer wieder auf ganz andere, für schweres Gelände konzipierte Fahrzeuge bezog, blieb nun zunehmend am Willys MA haften – nicht ohne aktives Zutun der Ursprungsfirma Willys Overland. In zahlreichen Werbekampagnen warb sie mit dem Image des Wagens als Held des globalen Konflikts. Darin tauchte das Fahrzeug in jeweils stark idealisierter Form in verschiedenen Situationen auf – von der Befreiung Frankreichs bis zu den Kampfhandlungen im Südpazifik. Auch wenn die Abbildungen mit der Realität der US-amerikanischen Kriegseinsätze wenig zu tun gehabt haben dürften, schrieben sie doch das Image des Jeeps als integraler Bestandteil dieser Kriegsführung fest.

**Abb. 4:**
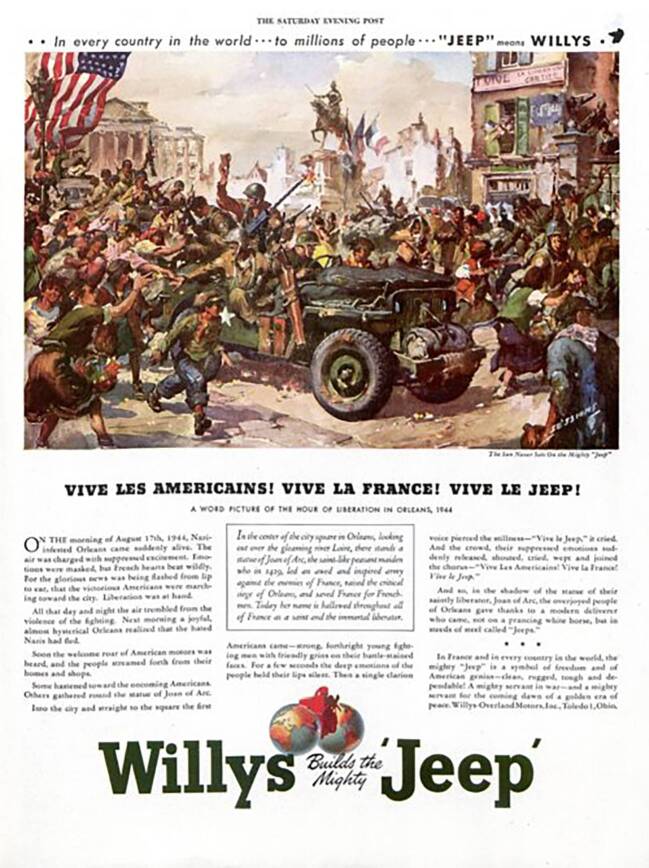
Die GIs bei der Befreiung Frankreichs (*The Saturday Evening Post*, 09.12.1944)

Bereits diese Entstehungsgeschichte verweist auf ein Charakteristikum des Autos, das es gerade auch hinsichtlich seiner späteren Verwendungen prägen sollte: Der Jeep war von Anfang an kein homogenes Automodell. Es handelte sich vielmehr um ein Bündel von Technologien, die in Hinblick auf die Notwendigkeiten der Rüstungssituationen der Jahre 1940 und 1941 auf eine sehr spezifische Rationalität hin ausgerichtet und noch dazu vom Auftraggeber gegen die Intention der beteiligten Firmen quasi *open-source* gestellt und in einer Art Konsortiallogik entwickelt wurden. Diese Logik war für das US-Militär bei solchen Rüstungsvorhaben nicht unüblich, in Bezug auf das spezifische zivile „Nachleben“ des Jeeps verdient sie allerdings besondere Beachtung. Der spätere Jeep war Resultat der Arbeit eines Netzwerks, das neben privatwirtschaftlichen, militärischen und politischen Interessen auch durch die Materialität des Fahrzeugs bestimmt war: Motor und Schaltung waren der schwere Kern des neuen Projekts; sie zu bauen band umkämpfte Ressourcen und erforderte auch politische Prioritätensetzungen.

## … zum Ladenhüter: die Suche der neuen „relevanten Nutzer:innengruppe“ für den alten Jeep

Der Kriegsheld der Werbung verlor in der unmittelbaren Nachkriegszeit viel von seinem Nimbus des Soldatenfreundes. Die Stückzahlen von nun „arbeitslos“ gewordenen Jeeps waren hoch, ihre Verwendung außerhalb des Kriegskontexts kaum geklärt. In neuen Werbekampagnen versuchte Willys die übriggebliebenen Fahrzeuge zunächst als neue Allrounder zu verkaufen, deren Stärke insbesondere in der Landwirtschaft in abgelegenen Regionen läge (vgl. etwa nachfolgende Quelle). Der Jeep sollte dorthin kommen, wo keine Feldwege hinführten, wo die Traktorenhändler von John Deere, der Hanomag oder Someca keine Handelspartner hatten, wo aber auch nicht das nötige Geld vorhanden war, um sich einen echten Traktor leisten zu können. Das Unternehmen bewarb diese neue Verwendung im landwirtschaftlichen Kontext sehr aktiv, wovon unzählige Werbekampagnen in den USA zeugen. Dazu gehörte nicht nur die Demonstration technischer Adaptionsfähigkeit, sondern auch die Integration in den sozialen Kontext abgelegener ländlicher Regionen – abseits der bereits voll mechanisierten Großbetriebe des Mittleren Westens (Kline 2000: 55–86).

**Abb. 5:**
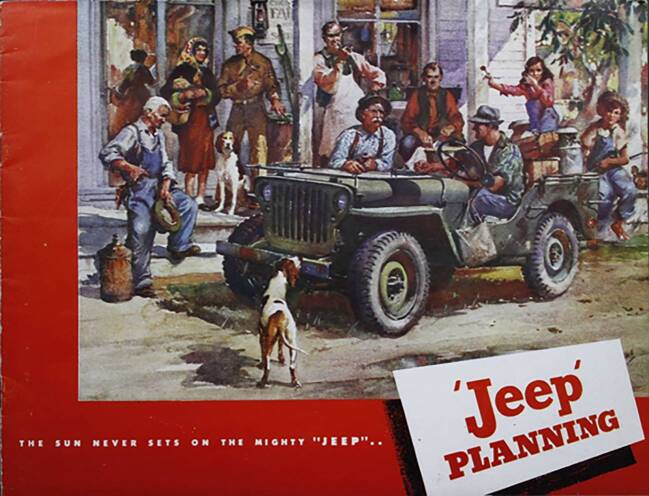
Titelseite eines Werbeprospekts von 1945 (Hagley Digital Archives, Taylor Vinson Collection of Transportation Ephemera, Series 1 Automobile Makes Box 1‑932, „Jeep Planning, 1945“)

Doch ein ähnlicher Massenerfolg wie im Krieg stellte sich nicht ein. Schon 1943 hatte die US-Regierung gemeinsam mit der National Automobile Dealers Association festgelegt, dass die riesigen Mengen von Armeefahrzeugen behutsam in den US-amerikanischen Markt integriert werden sollten, um die übrige Produktion nicht zu gefährden.^2^ Die Gefahr, dass der Markt durch die massive Schwemme ausgemusterter Militärfahrzeuge blockiert würde, schien real. Im Idealfall sollten ehemalige Soldaten „ihre“ Jeeps gleich mit nach Hause bringen, weshalb sie ihnen zu Kosten weit unter dem Marktpreis angeboten wurden.^3^ Interessierte Veteranen mussten nachweisen, dass sie in der Landwirtschaft tätig waren oder den Jeep für andere geschäftliche Tätigkeiten brauchten (insbesondere Handwerk). Auch dies galt nur in Verbindung mit einer Bestätigung des regionalen Armeebüros, der Small War Plants, wo sie von kriegsversehrten Veteranen im Gebrauch eines Jeeps unterwiesen werden sollten.^4^

Die Nutzung von Automobilen in den ländlichen Gebieten der USA, auch als landwirtschaftliches Werkzeug einer Protomechanisierung, war dabei keineswegs eine Neuheit. Wie Kline und Pinch gezeigt haben, ging diese nicht intendierte Verwendung von Automobilen bis an den Anfang des Jahrhunderts zurück, wo die Farmer des Mittleren Westens sich nach anfänglicher Zurückhaltung und Ablehnung die neuen Autos und insbesondere den Ford T schnell als lokalen und transportablen Kraftgenerator aneigneten. Für Pinch und Kline war dies der „re-opener“ einer an diesem Punkt bereits abgeschlossenen Technologie (Kline & Pinch 1996). Auch Ford machte sich diesen alternativen Nutzen des Autos jenseits des reinen Fortbewegungsmittels zunutze und bewarb am Anfang des 20. Jahrhunderts das sogenannte „conversion kit“ – eine Vorrichtung zur erleichterten Verwendung des Automotors für landwirtschaftliche Techniken wie Sägen, Dreschen oder auch das Verbuttern von Milch (ebd.). Doch ab den 1920er Jahren waren solche Aushandlungen der Nutzung des Automobils durch die relevanten sozialen Gruppen der ländlichen Gesellschaft zunehmend abgeschlossen. Der Ausbau der Verkehrsinfrastruktur erschloss den ländlichen Raum, lokale Gemeinschaften gewöhnten sich an die veränderten räumlichen Relationen, und die Mechanisierungsoffensive von Roosevelts New-Deal-Politik stattete in den 1930er und 1940er Jahren eine immer größere Zahl von Farmern mit leistungsstarken Landmaschinen aus (Volti 2004: 46; Clarke 1991). Die Notwendigkeit für Jeeps als Allround-Behelfsmaschinen bestand kaum noch.

Aus diesen Gründen hätte es der restriktiven Handhabung der Armeeführung bei der Vermarktung der ausgemusterten Jeeps gar nicht bedurft, denn bereits nach kurzer Zeit stellte die beteiligte Vereinigung der Autohändler NADA resigniert fest: „[I]t appears that the demand for surplus Jeeps from governmental agencies and veterans has not been as heavily as anticipated.“^5^ Der Absatz des ehemaligen Militärgutes im landwirtschaftlichen Kontext verlief also schleppend. Trotz all ihrer Beliebtheit im Krieg erwiesen sich die Jeeps in Friedenszeiten als Ladenhüter: Für die großen Flächeneinheiten des Mittleren Westens waren sie zu klein und die Traktionskräfte des Fahrzeugs bei den hohen Drehzahlen zu niedrig für wirklich intensive Feldarbeit. Zwar war der Jeep nicht etwa unbeliebt, doch der Absatz als Universalmaschine blieb eine Chimäre. Ein Großteil der Jeeps wurde eben nicht auf dem Land verwendet, sondern von Handwerker:innen, die lediglich in Gegenden ohne eigenes Stromnetz den Stromgenerator benötigten (Anonym 1945: 102ff.).

## Der Jeep als (post-)koloniale Infrastruktur

Mit dem relativen Misserfolg bei der Eroberung eines zivilen Massenmarktes stellte sich auch die Frage nach eventuell anderen Nutzungen und entsprechenden Nutzer:innengruppen. Um weder die Gefahr eines zusammenbrechenden US-Automobilmarkts weiter zu befeuern noch die Rüstungsgüter der U.S. Army angesichts einer unklaren Bedrohungslage vollständig aus der Hand zu geben, waren viele der Jeeps einfach in den verschiedenen Einsatzorten des US-Militärs zurückgelassen worden, wo sie zunächst zivil genutzt werden konnten (Seelinger 2006: 13). Verbunden blieb dies für die US-amerikanischen Militärs allerdings mit einem gewissen Risiko, denn nicht selten wurden die Jeeps als Symbole von technologischer Überlegenheit zum begehrten Objekt für Diebe (Kim 2016: 70). Einen Jeep von der Armee zu bekommen oder ihn auf halb- oder gänzlich illegalem Wege zu übernehmen, wurde in vielen Ländern, in denen die US-Amerikaner im Lauf des Kriegs aktiv gewesen waren, zu einem neuen Statussymbol. Wie im Folgenden zu sehen sein wird, koinzidierte die Umnutzung der ursprünglich militärischen Gerätschaften mit der Erosion kolonialer Machtverhältnisse und verstärkten entwicklungspolitischen Maßnahmen. In vielen Regionen des globalen Südens fand der Jeep schnell seine Rolle im Aufbau neuer substaatlicher Räume und territorialer Zusammenhänge in früheren oder noch kolonisierten Gebieten.

Die Erschließung kolonialer Räume durch entsprechende Infrastrukturprojekte war im frühen 20. Jahrhundert geprägt gewesen von Projekten des Eisenbahnbaus (Aguiar 2011). Helen Tilley beschreibt, dass es in diesen Projekten bei Weitem nicht nur um neue Transportwege ging. Nach ihrer Lesart spiegelte die Form infrastruktureller Erschliessung der kolonialen Territorien auch ein ganz anderes Verständnis der Kolonien als „development states“, in denen die Erreichbarkeit der Bevölkerungen im kolonialen Hinterland insbesondere mit deren Umformung in Produzent:innen von Gütern für internationale Märkte einherging (Tilley 2011).^6^ Die Eisenbahn definierte auch die sozioökonomischen Beziehungen zwischen Kolonisierenden und Kolonisierten um.

In der spätkolonialen Zeit veränderten sich die Ambitionen hinter dieser Durchdringung der kolonialen Räume. Die Straße trat in Konkurrenz zur Eisenbahn beziehungsweise ergänzte diese, denn eine wirklich flächendeckende Durchdringung der kolonialen Räume mit Eisenbahnnetzen nach europäischem Vorbild blieb eine Utopie (Pirie 2011: 45; Tshund’olela 1987: 237–241; Heap 1990: 19–37; Turton 1997: 1–15). Gleichzeitig blieb die verhaltene Motorisierung der Kolonien ab den 1920er Jahren ein elitäres Projekt, das etwa die räumliche Überlegenheit europäischer Siedler:innen gegenüber der lokalen Bevölkerung festigte (Clarsen & Veracini 2012: 889f.; Gewald 2002: 257–286; Alber 2002: 79ff.; Green-Simms 2017: 31–58). Der Kampf um ausreichende Mittel für einen effizienten Straßenbau, der nicht nur als Versorgungsweg rechts und links der wenigen etablierten Eisenbahntrassen diente, nahm breiten Raum im Forderungskatalog vieler Kolonien gegenüber den Kolonialmächten ein, wie Jennifer Hart am Beispiel der Gold Coast unter britischer Kolonialadministration betonte (Hart 2016: 33; Guldi 2012). Sie stand für die Ausweitung der Automobilität auf große Teile der Bevölkerung (Havik 2009: 48–74), denn nur durch Investitionen in die Infrastruktur war es möglich, von der *off-trail*-Logik der spätkolonialen Motorisierung wegzukommen und etwa durch billigere Fahrzeugtypen in den Kolonien Mobilität für breitere Bevölkerungsgruppen erreichbar zu machen.

Gleichzeitig überspannten diese Technopolitiken den anscheinend so harten Bruch zwischen der spät- und der postkolonialen Periode. Automobile Erreichbarkeit abgelegener Räume war ein Anliegen, das die spätkolonialen Infrastrukturpolitiken und die im Entstehen begriffenen Nationen der Phase nach der Dekolonisierung teilten. Dieser neue technische Möglichkeitsraum befeuerte sowohl die Vorstellung der Ausweitung von kolonialen Regierungspraktiken als auch die Vorstellungen der Durchdringung des eigenen nationalen Raums – ein Kernanliegen der entstehenden Unabhängigkeitsbewegungen. Zunehmend wurden diese Anliegen aber auch durch einen dritten Akteur bestärkt, denn sie hatten auch in den US-amerikanischen Unterstützungspolitiken der Nachkriegszeit einen festen Platz. Die Entwicklung neuer, alternativer Formen des Ausbaus technischer Infrastrukturen war dabei ein fester Teil des Wiederaufbauprogramms in Europa und wurde von hier aus für andere Teile der Welt passend gemacht (Adalet 2018; Seely et al. 2004: 236; Mom 2020). In diesem Kontext stand Automobilität für eine neue spontane Infrastruktur, die nicht mehr auf die absolute Durchdringung des Raums und auch nicht mehr auf die Kopie von westeuropäischen Raumkonzepten, sondern auf eine ubiquitäre und dennoch Ad-hoc-Erreichbarkeit von abgelegenen Orten und Bevölkerungsteilen abzielte. Der Jeep schien genau diesem Anliegen zu entsprechen, denn er löste Automobilität von der Existenz eines festen Straßennetzes. Das Fahrzeug war nicht angewiesen auf große Investitionsprogramme oder auf die ressourcenintensiven Eisenbahnbauten; es war unabhängig von „politischen Halteapparaten“ einer groß angelegten Modernisierungspolitik (Guldi 2012).

Um diese These des Jeeps als eine spontane und mobile Infrastruktur zu erhärten, soll der Blick auf die Rolle des Jeeps in verschiedenen regionalen Kontexten dieser Übergangsphase gelenkt werden:

1. Gerade in den west- und nordafrikanischen Teilen des französischen Kolonialreichs war es bis in die spätkoloniale Phase kaum zu einem Ausbau von Bahnlinien und Straßennetz gekommen. Nur circa zwölf Prozent des knapp 100.000 Kilometer umfassenden französischen Eisenbahnnetzes befanden sich in den französischen Kolonien, obwohl diese über 90 Prozent des kolonialen Frankreichs ausmachten (Bancel 2022: 503–508). Die Versuche, dieses Netz weiter auszubauen, scheiterten einerseits an dem politischen Grundsatz, die Entwicklung der Kolonien nur auf deren eigene Kosten – also ohne Schuldenaufnahme – zu finanzieren, wodurch bis zur Einrichtung des Investitionsfonds für die Kolonien (FIDES) 1946 kaum die nötige Finanzierung für ein solches Unternehmen zur Verfügung stand. Andererseits verliefen solche Bemühungen aber auch wortwörtlich im Sand. Schienen wie auch Straßen konnten technisch kaum in den instabilen Wüstensand der Sahara und der Sahelzone gebaut werden. Unterfangen, eine Schienenverbindung zwischen den französischen Kolonien in Nordafrika und der westafrikanischen Küste herzustellen, scheiterten schon nach wenigen hundert Kilometern (Mom 2020: 133–152). Umso gewichtiger war der Versuch, mithilfe von Geländefahrzeugen die Wüste von Nord nach Süd zu durchqueren. Am 7. Januar 1923 gelang dies erstmals fünf Raupenfahrzeugen von Citroën in 22 Tagen. Sie bildeten den Auftakt zu einer regen Expeditionsaktivität in der Wüste, deren Ziel in erster Linie die Demonstration technischer Machbarkeit (und damit zivilisatorischer Überlegenheit der Kolonialherren) war. Ein realer Nutzen war mit der Durchquerung der Sahara weder im wirtschaftlichen noch im militärischen Sinne verbunden (Thomas 1953: 327–339; Pirie 2011: 41). Diese Überlegenheit lag nun nicht mehr in der Effizienz des Straßenbaus, der aufgrund des Wüstensandes nicht stattfand. In den Sandwüsten mussten eingefahrene Spuren vermieden werden und Fahrzeuge konnten sich daher vor allem auf teils hunderte Meter breiten Pisten fortbewegen (Krais 2019: 143–158). Stattdessen war es allein die Potenz der Fahrzeugtechnologie, nicht etwa schnelle Fahrzeuge herzustellen, sondern solche, die Benjamin Thomas als „colonial vehicles of high clearance and durable construction“ bezeichnete – häufig Halbkettenfahrzeuge – und die in der Lage waren, das Gelände zu meistern (Thomas 1953: 327–339). Diese frühen Geländefahrzeuge waren allerdings für einen umfassenderen Einsatz zu kosten-, ressourcen- und wartungsintensiv, weshalb sie lange nur zu punktuellen Anlässen – etwa besagten Saharaexpeditionen – eingesetzt wurden. Mit dem neuen Getriebe des Jeeps wurden diese „colonial vehicles“ behäbigerer Art abgelöst durch die hochtourigen Motoren, die im Sand auch eine höhere Geschwindigkeit erreichen konnten. Der Jeep wurde zum Fahrzeug einer „second colonial occupation“. Für die Französ:innen war dies eine wichtige Motivation, den Autoproduzenten Delahaye mit einer eigenen Version des Jeeps zu beauftragen, die 1951 bei der „Rallye Méditerranée-Le Cap“ gerade in Hinblick auf die Wüstentauglichkeit vorgestellt werden sollte. Die Präsentation des Rallye-Projekts verdeutlicht dabei die Ambitionen, die hinter dem Einsatz der neuen Technik lagen: „Das Ziel der Organisatoren liegt weniger im sportlichen Charakter der Veranstaltung, als darin, durch ein spektakuläres Ereignis die Aufmerksamkeit auf Afrika als Element einer neuen weltumspannenden Politik und Strategie zu lenken. Hierdurch sollen die Perspektiven auf Afrika verändert werden, um damit engere Beziehungen in allen Bereichen vorzubereiten: in der Wirtschaft, im Tourismus, in der Kultur.“^7^ Die Fabrikation von Delahaye zeichnete sich allerdings durch zahlreiche technische Schwächen aus, die erst mit der Produktion des M 201 durch Peugeot-Hotchkiss ab 1955 behoben waren. Auch von diesem Wagen wurde umgehend eine Wüstenversion gebaut, die sich durch verstärkte Federung und Chassis auszeichnete (Boniface 2005; Decker 2002a; Decker 2002b).
2. Die spontane Durchdringung von kolonialen Räumen abseits von Schienen und Straßen war nicht ein Zweck an sich. Jeeps verbanden Kommunikationsräume unabhängig von den teils nur rudimentär ausgebauten nationalen Medienlandschaften in den Kolonien. Von nationalen Agrar- oder Gesundheitsministerien, aber auch von den entstehenden internationalen Organisationen wurden Jeeps, sofern vorhanden, gerade auch als Teil von Aufklärungs- und Informationskampagnen eingesetzt. Ab den frühen 1950er Jahren brachten sie nicht nur Agrar- und Gesundheitsfachleute in entlegene Dörfer, sondern zunehmend auch Aufklärungsfilme, die mit den auf dem Dach montierten Projektoren und den neuen *power outtakes* (fester Bestandteil des Willys Overland Nachkriegsmodells) in den Dörfern vorgeführt werden konnten.

Gerade das US-amerikanische Militär bemühte sich in der Nachkriegszeit aber auch um einen Einsatz jenseits dieser funktionalen Logik und sandte die Jeeps regelmäßig in die Dörfer, um die neuesten Hollywood-Kassenschlager vorzuführen (Virilio 1989; Trumpbour 2002). Für das von US-Truppen im UN-Auftrag kontrollierte Südkorea in den 1950er Jahren paraphrasiert Han Sang Kim: „While city cinemas fulfilled the role of a strategic foothold to lure the urban audience, itinerant film exhibitions were active primarily in efforts to reach the everyday lives of people in the countryside directly and thus to mobilize the rural audience“ (Kim 2016: 63–85). Er beschreibt, wie hierdurch der Jeep zu mehr als einem bloßen Vehikel wurde. Als Symbol der Anbindung abgelegener ländlicher Gebiete an das durch unmittelbare Kommunikationsakte entgrenzte *global village* wurde der Jeep zu einer Technologie der Modernisierung.

3. Über solche Programme gewann der Jeep seine Stellung als eigentliche Infrastruktur der Modernisierung, denn er ermöglichte dauerhaften Zugang zu bestimmten Gegenden. In der Nachkriegszeit häuften sich die Momente, in denen die Technologie des Jeeps nicht mehr als ein Werkzeug für den Aufbau anderer Programme erschien, sondern vielmehr solche Programme gestaltete. So benannte die von Nelson A. Rockefeller gegründete American International Association for Economic and Social Development (AIA) Ende der 1940er Jahre „A man, a girl, and a jeep“ (Pernet 2023: 267–270) als die wesentlichen Bestandteile, deren man bedürfte, um ländliche Entwicklung in Gang zu setzen. Gemeint waren damit ein Agrarexperte, eine (meist junge) Beraterin für ländliche Hauswirtschaft sowie der Jeep als das adäquate technische Medium, um die Inhalte der Entwicklungsprogramme in die Dörfer zu bringen. Dabei ging es nicht nur um das richtige Fortbewegungsmittel, sondern der Jeep wurde zum handelnden Ding – im latour’schen Sinne zum Aktanten – einer solchen Modernisierungsgeschichte, denn die technischen Vorgaben bestimmten auch die Formen und Inhalte der Programme mit. So definierten seine Reichweite, seine Ladekapazitäten und seine Wartungsanfälligkeit die Grenzen von Entwicklungsprogrammen. Gleichzeitig war das Skript des Fahrzeugs schnell veränderbar: Die technischen Features aus dem militärischen Kontext, etwa der mobile *power outlet*, konnten ebenso gut für die Vorführung von Lehrfilmen wie für das Zersägen von Hindernissen im Feld benutzt werden.

Besonders deutlich wurde die eigene Handlungsmacht der Technik bei den Entwicklungsprogrammen der US-Regierung im Gefolge von Trumans Point-Four-Programm von 1949: Für alle US-amerikanischen Organisationen, die sich mit der Umsetzung von US-amerikanischen Programmen beschäftigten (beginnend mit der Foreign Operations Administration FOA), war die Ausstattung dieser Projekte mit Jeeps einer der ersten Umsetzungsschritte – so etwa bei den ab den frühen 1960er Jahren proliferierenden globalen Familienplanungsprogrammen. Im türkischen Fall war die Lieferung von zunächst 50 Jeeps für das Programm (ein paar Jahre später folgten weitere 1.400) die Kernforderung, welche türkische Akteur:innen an die US Agency for International Development (USAID) stellten.^8^ Die Jeeps stellten nicht Werkzeuge für die Erreichung eines Programmziels dar, sondern waren in vielerlei Hinsicht selbst Programm: Ihre Fähigkeit der kommunikativen Verbindung in entlegene Regionen war letztlich auch das Herzstück des Programms zur Familienplanung. Gerade in diesen heiklen Missionen, in denen die Frage von nationaler Souveränität sich mit den Fragen von Geschlechtergerechtigkeit überschnitt, traten nicht etwa internationale Expert:innen in Erscheinung. Im türkischen Fall legten die Akteur:innen besonderen Wert darauf, dass ein solches Programm zumindest nach außen hin streng in türkischer Hand blieb. Stattdessen war es der vermeintlich neutrale Jeep, der Ärzt:innen aus der Region, Hebammen aus den lokalen Gesundheitszentren und Informationsmaterialien des türkischen Gesundheitsministeriums in entlegene Dörfer transportierte. So war der Jeep für viele lokale Bevölkerungen der einzige internationale Akteur, der in einem solchen Programm in Erscheinung trat (Hartmann 2020: 364–377).

4. So sehr der Jeep auch als Repräsentant der Moderne wahrgenommen wurde: Er brachte keineswegs nur Neues, sondern affirmierte auch Altes. Wie schon oben angesprochen waren die meisten Menschen, die mit dem Jeep in die Dörfer kamen, keineswegs vollkommene Unbekannte, sondern es handelte sich um Berater:innen, Hebammen, Ärzt:innen oder Lehrer:innen, die auch vorher für die Dörfer zuständig gewesen waren. Sie kamen nun einfach öfter. Und auch auf anderer Ebene affirmierte der Jeep eher soziale Strukturen als sie neu zu erschaffen. Dort, wo der Jeep auf lokale Akzeptanz und Unterstützung angewiesen war, wurde nochmals sichtbar, dass er nicht einfach nur ein Werkzeug war.

Am Beispiel des syrischen Falls argumentiert Mehdi Sakatni gegen eine Lesart, in der die Durchdringung der bis anhin durch den europäischen Kolonialismus unerschlossenen Räume gleichbedeutend gewesen sei mit der Verdrängung traditioneller Gesellschaftsformen. Er weist für die Nomad:innen während der französischen Mandatszeit nach, dass diese bei Weitem nicht nur Empfänger:innen neuer Technologien waren, deren transformativer Kraft sie tatenlos zusahen (Sakatni 2019: 159–169). Vielmehr machten sich viele Beduin:innen die neue Technik zur Bestätigung und Erhaltung ihrer sozioökonomischen Strukturen zunutze. Sie dienten nicht nur einer effizienteren Viehhaltung über lange Distanzen, sondern erlaubten auch Machtdemonstrationen und konkrete Überfälle auf konkurrierende Stämme. Entsprechend waren viele *sheiks* schon früh daran interessiert, sich ein solches Fahrzeug zu sichern, ohne dass hierdurch Kamele oder Pferde unnötig geworden wären. Die verschiedenen Transportsysteme existierten nebeneinander und in gegenseitiger Abhängigkeit. Jeeps fuhren nicht nur im Auftrag der Modernisierungsprogramme des Kalten Krieges. Sie ebneten den Weg auch in multiple Modernen oder vielmehr in das, was Gijs Mom gerade in Hinblick auf die Mobilitätsgeschichte als „layered development“ bezeichnet (Mom 2020: 384–574), in der das Nebeneinander von alten und neuen Technologien und ihre lokale Aneignung entscheidende Charakteristika waren.

## Design: zur Interdependenz neokolonialer und neoliberaler Raumvorstellungen

Die Perspektiven auf den Jeep als eine spät- und postkoloniale Infrastruktur der Modernisierungspolitik scheinen sich kaum in einen Zusammenhang mit dem bringen zu lassen, was die kommerziell eigentliche Erfolgsgeschichte des Jeeps in der Nachkriegszeit (und gerade auch bis heute) ausmachte. In großen Mengen verkauft wurde das Fahrzeug nicht als landwirtschaftliches Gerät und auch nur begrenzt als Transportgerät für Entwicklungsprogramme (hier blieben die tatsächlich verwendeten Stückzahlen, insbesondere an neu produzierten Wagen, überschaubar), sondern ab den 1950er Jahren zunehmend als Statussymbol in (zunächst US-amerikanischen) Vorstädten. Es scheint, als sei eine größere Diskrepanz zwischen diesen Nutzungsformen und den damit verbundenen Nutzer:innengruppen kaum möglich. Dennoch möchte ich dafür plädieren, beide Phänomene der Verwendung von Technik nicht voneinander losgelöst zu verstehen. Obgleich die wirtschaftliche Vermarktung des Fahrzeugs von großen Brüchen geprägt war, nahm das Marketing doch genau die Bedeutungsdimensionen auf, die sich der Jeep in seinen globalen Verwendungsformen angeeignet hatte, wie im Folgenden zu zeigen sein wird.

Da der Bedarf an Jeeps durch die nicht mehr verwendeten Armeebestände zunächst durchaus gedeckt war, kam der Produzent des Fahrzeugs zunehmend ins Schlingern. Willys hatte ab 1946 versucht, seine gesamte Produktionskapazität auf den Jeep zu verwenden, auch nach dem Ende des Krieges. Im selben Jahr machte sich auf dem US-Markt eine weitgehende Rohstoffknappheit breit. Willys nutzte den verfügbaren Stahl prioritär für den Jeep, der durch sein Gewicht und den schweren Motor relativ ressourcenaufwendig war. Alle kleineren Modelle des Autobauers mussten hintanstehen. Dies galt insbesondere für den Bau eines eigentlich geplanten neuen Pkw-Modells (Livingston 1947). Eine solche strategische Entscheidung machte das ganze Unternehmen von Erfolg und Misserfolg des Jeeps und damit auch von der Akzeptanz durch neue Nutzer:innen abhängig.

In seinem Versuch, den Jeep und damit das dahinterliegende kommerzielle Unternehmen zu retten, wechselte das Fahrzeug noch einmal die Nutzer:innengruppe – diesmal unterstützt von weitgehenden Anpassungen in seinem Design und äußeren Erscheinungsbild, denn er wurde vom Armee- zum Mittelklassewagen. Willys setzte in seinem neuen Marketingkonzept in erster Linie auf den Designer Brooks Stevens, einen der namhaftesten Industriedesigner der US-amerikanischen Nachkriegszeit (Caspers 2018). Stevens war allerdings nicht dafür berühmt, industrielles Design durch neue Formen zu beleben, sondern eher technologische Innovation in einem retrospektiven Äußeren zu verhüllen. Stevens hatte schon in den frühen 1940er Jahren prognostiziert, dass das Nachkriegsdesign in etwa dem Vorkriegsdesign entsprechen würde und damit die reaktionären Konsummuster der US-Gesellschaft zutreffend vorhergesagt (Adamson 2003): Die zahlreichen und radikalen Veränderungen in der Weltordnung und in der alltäglichen Technologieabhängigkeit würden dazu führen, dass Verbraucher:innen eher nach alten Formen suchen würden, die ein gewisses Maß an Sicherheit versprachen. Dies galt auch für das neue Design des Willys Station Wagon, der auf Grundlage der Jeeptechnologie entwickelt worden war. Letztlich bestand der Station Wagon aus dem Chassis des alten MA-Modells, umgeben von einer Karosserie, die in ihren Formen eher an eine Pferdekutsche als an ein modernes Mehrzweckgerät erinnerte.

**Abb. 6:**
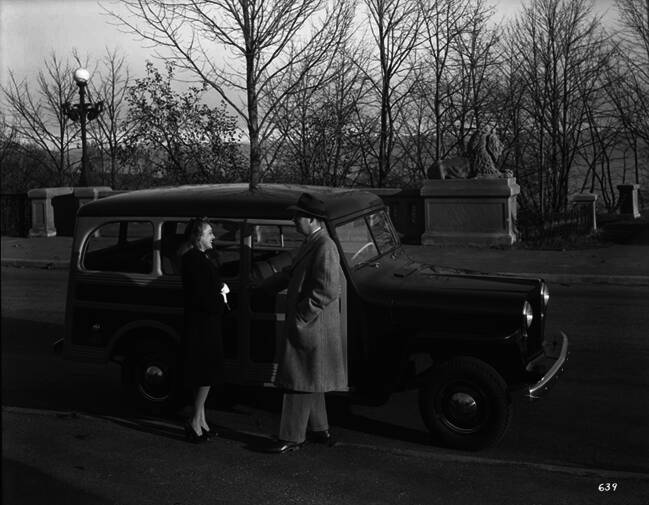
Brooks Stevens zusammen mit einer unbekannten Frau vor dem Willys Overland Station Wagon, 1946 (Milwaukee Art Museum, Brooks Stevens Archive, BSA_0639)

Der Alleskönner sollte nun den Transport mittelgroßer Gruppen oder eben mittelschwerer ziviler Lasten ermöglichen. Doch auch hier galt: Fallende Geburtenraten und Familiengrößen in der US-amerikanischen Mittelschicht, die auch nur theoretisch am Kauf des Jeeps hätte interessiert sein können, liefen solchen Transportnotwendigkeiten entgegen (Heinemann 2018: 164–186). Und mit dem expandierenden Straßenbau in den USA, mit der Verfügbarkeit von elektrischem Strom in den entlegensten Winkeln fiel auch der eigentliche Bedarf für ein solches Universalfahrzeug mehr und mehr weg. Die Idee der infrastrukturellen Durchdringung wurde immer weniger relevant. Nochmals auf die Spitze trieb Stevens diese Verknüpfung von Raumbeherrschung und retrospektivem Design beim neuen sportlichen Modell Jeepster.

Um den Jeep zu retten, reichte die Fokussierung auf die technischen Fähigkeiten des Fahrzeugs bald nicht mehr aus. Und Willys selbst war nicht mehr in der Lage, mit einem vollständigen Wechsel des Marketings die Absatzzahlen zu stabilisieren. Für den Konzern verfinsterte sich Anfang der 1950er Jahre die Lage, was 1953 zum Verkauf von Willys Overland an Henry J. Kaiser führte.

In den USA war in der Zwischenkriegszeit eine verwirrend unübersichtliche Landschaft von kleinen Automobilbauern gewachsen. In der Nachkriegszeit sortierte sich dieses Gewirr schnell. Kaiser hatte bereits einen Mischkonzern aufgebaut, der sich vor allem vertikal konzentriert hatte und dabei etwa auf die Synergien aus der gemeinsamen Rohstoffbewirtschaftung setzte (Foster 1985: 1–23). Besonders groß war Kaiser mit dem Bau von Schlachtschiffen, insbesondere mit der Klasse der Liberty ships, während des Krieges geworden (vgl. Adams 1991; Heiner 1991; Heinrich 2020: 216–225). Doch der Name von Henry Kaiser war seit den 1930er Jahren auch untrennbar mit dem Ausbau der US-amerikanischen Infrastruktur verbunden, denn er war einer der fünf führenden Industriellen hinter einem Konsortium, das etwa den Bau des Hoover-Damms als erster Großstaudammanlage mit moderner Stahlbetontechnik betrieben hatte (Wolf 1996; Tassava 2003; Foster 1989).

In gewisser Weise war diese infrastrukturelle Erneuerung der USA Kaisers Thema, dem er die verschiedenen Geschäftsbereiche seines Unternehmens unterordnete: Während des Krieges hatte er sich einen Namen gemacht mit seiner Lobbyarbeit für den Ausbau der Verkehrsinfrastruktur. Durch neue Straßen und Highways sollte das Land auch abseits der Städte erreichbar werden. Wohnräume gerade für die wachsenden Mittelschichten sollten erschlossen werden. Diese Durchdringung der *suburban frontier* war sein erklärtes Ziel. 1944 veröffentlichte er einen großen Plan für ein nationales Flughafennetz, das mit den Highways an die großen Städte angebunden war. Ziel sollte ein Land der Erreichbarkeit sein, schnell und einfach pendelbar.^9^ Diese Allianz aus liberalem Unternehmertum und dem durch die New Deal-Politik geprägten Infrastrukturausbau überspannte die Brüche des Zweiten Weltkriegs in den USA (Smith 2006: 232–257).

Die Suche nach einer neuen Form von liberalem Individualismus bildete die Klammer, die Kaisers Visionen mit dem Jeep verband. Ein freier männlicher Eroberer war der Held der postkolonialen und der suburbanen *frontier*. Doch statt frei durch die Natur zu fahren, war es in den USA das Ideal, schnell über große Distanzen mobil sein zu können, was man mit Cotten Seiler einen „Highway-Individualismus“ nennen könnte (Seiler 2008: 81–104; auch Rollins 2006: 693ff.). Der Jeep schien dabei seinen Nutzer mit der Welt zu verbinden, denn er stand nicht nur für Werte wie Unabhängigkeit und Erreichbarkeit, sondern auch Mündigkeit und Teilhabe an einem demokratischen Kommunikationsraum – auch dies eng verbunden mit Marshall McLuhans „global village“. Die Selbstverwirklichung dieses „Jeep-Individualismus“ sollte es sein, die den mündigen (und immer noch männlichen) Staatsbürger in die Lage versetzte, seine unternehmerischen Kreativkräfte bestmöglich zu verwirklichen und dabei gleichzeitig für die eigene Sicherheit und die der von ihm angeführten Familie zu sorgen. Die Bedrohung durch starke soziale Ungleichheit wurde kompensiert durch die Partizipation an neuen Konsumwelten (Cohen 2004: 112–165). Der geschützte Raum des großen, von vornherein überdimensionierten Jeeps oder Station Wagons schien dies in besonderem Maße zu gewährleisten.

Das Design, das Brooks Stevens dem zivilen Jeep gegeben hatte, nahm diese Werte auf. Das universell einsetzbare Fahrzeug vermarktete auch Kaiser im „Retrolook“. Und doch war die Formensprache lokal schnell anpassbar, denn der Jeep war keineswegs einheitlich. Zahlreiche Produktionsschritte von Motor, Karosserie und Chassis blieben ausgelagert. Eine einheitliche Konstruktion konnte das Unternehmen nicht durchsetzen, vielmehr blieb der Jeep ein dezentrales Projekt. Lokale Firmen weltweit konnten mit der Produktion von einzelnen Blechteilen beauftragt werden. Sehr verschiedene Formen des Jeeps in verschiedenen Teilen der Welt waren das Ergebnis. Ab 1956 produzierte etwa Industrias Kaiser Argentina einen guten Teil der Jeeps der Reihe CJ mit einem eigenen angepassten Design. Ähnliche Tochterfirmen gab es in verschiedenen südamerikanischen Ländern – etwa in Brasilien, wo sie später von großen Autokonzernen wie Ford oder Renault aufgekauft wurden. Auch in dieser kommerziellen Dimension und der Flexibilität des Designs nahm der Jeep damit eine Scharnierfunktion in einer neuen, sich globalisierenden Warenwelt ein und stand gleichzeitig für das Bedürfnis wachsender Mittelschichten nach Sicherheit, die durch keine Technologie so gut verkörpert werden konnte wie durch den ehemaligen automobilen Kriegshelden.

## Fazit

Die parallele Karriere des Jeeps einerseits als postkoloniales Fahrzeug, das eine der wichtigsten Infrastrukturen der Modernisierungsphase darstellte, und andererseits der neuen Rolle für eine US-amerikanische Mittelschichtsmoderne, die auf individuelle Mobilität und rückwärtsgewandte kulturelle Bezüge setzte, bleibt schwierig zusammenzudenken. Gerade die Parallelität verschiedener Nutzungsformen weist aber darauf hin, dass mehr dahintersteckte als ein einfacher Wechsel der Marketingstrategie nach dem Ende des Krieges. Der Jeep verband den Anspruch auf die Durchdringung von spät- und postkolonialen Räumen im Rahmen der modernisierungstheoretisch inspirierten Entwicklungsprojekte der Nachkriegszeit mit dem Weg in eine vernetzte Moderne im neu entstehenden US-amerikanischen Suburbia.

Von Bruno Latour stammt der Vergleich unseres Verständnisses der Moderne mit einem Eisenbahnnetz: Es durchdringt riesige Räume. Wenn man aber näher hinschaut, sind es immer nur ganz vereinzelte Knoten, die Teil dieses modernen Netzwerks sind – die Bahnhöfe. Alles, was dazwischen liegt, verharrt in einer Art von vormodernem Status, in dem es auf unwegsamen Pfaden versucht, sich mit diesen vereinzelten Knotenpunkten (also mit den Bahnhöfen) in Verbindung zu setzen (Latour 1997: 158f.). Latour macht dies zum Bild für eine Moderne, die nicht stattfindet; die nur auf dem Papier universell ist. In der direkten Nachkriegszeit baute der Jeep sein Geschäftsmodell darauf auf, genau dieses Paradox aufzulösen. Er schien das Instrument darzustellen, eine neue technosoziale Wirklichkeit flächendeckend verfügbar zu machen – im Mittleren Westen wie auch in den spät- und postkolonialen Räumen des globalen Südens. In beiden Fällen blieb die scheinbare Unabhängigkeit des Jeeps allerdings auch eine Illusion, denn er war weiterhin angewiesen auf die Versorgung mit fossilen Brennstoffen und damit auf ein Tankstellennetz sowie auf Förderung und Transport des Kraftstoffs.

Beide Arten der Umdefinierung erwiesen sich dabei trotz ihres fiktiven Charakters als persistent und waren für die Stabilisierung der Technik des Jeeps viel nachhaltiger als der ursprüngliche militärische Nutzen (Pinch & Bijker 1987). Sie erreichten in Gestalt des SUV schliesslich eine Form von kommodifzierter Generalisierbarkeit (Caspers 2018). Der Siegeszug des Jeeps wurde in dieser Hinsicht in der Literatur als Ergebnis einer *consumer positivity* verstanden, die die US-amerikanische Gesellschaft in den vergangenen fünf Jahrzehnten mehr und mehr prägte. Die deutlich kritischen Untertöne einer solchen Analyse eines ausufernden Konsumverhaltens sind überzeugend und nachvollziehbar (McCarthy 2007; Bradsher 2002; Rollins 2006), doch nehmen sie ein Objekt wie den Jeep und die ihm zugrunde liegende Technik weder in der postkolonialen Dimension noch in der Wandelbarkeit der Technologie in der Interaktion mit verschiedenen Nutzer:innengruppen ernst. Wie Rollins angeführt hat, kann es der Konsumgeschichtsschreibung nicht darum gehen, Gründe für die Ablehnung bestimmter Produkte zu liefern, sondern vielmehr die verschiedenen Bedeutungsebenen zu erkennen, mit denen sich ein Produkt wie der Jeep und später der SUV verbindet und die alle Teil seines Erfolgs sind (Rollins 2006).^10^

In meiner Interpretation liegt der Übernutzung des Jeeps beziehungsweise des SUV mehr zugrunde als die Tatsache, dass es sich hier um einen entsprechend teuren Luxusgegenstand handelt. Die Einschreibung von Modernisierungsideologien einer postkolonialen Welt spielen eine Rolle in der Bedeutungszuschreibung durch heutige Nutzende. Der Jeep erschuf die Illusion einer Moderne, die durch Kontakt und Kommunikation räumliche Weite überspannen konnte. Er brachte nicht nur Expertise und Informationen, sondern auch neue Vernetzungsmöglichkeiten mit, da er Kommunikation ermöglichte, wo diese sonst kaum stattfand. Das *global village *war nicht nur mittels Zeitungen und Radios verbunden, sondern materiell eben auch durch den Jeep. Gleichzeitig hegte das sichere Innere des Fahrzeugs die Insass:innen in dieser globalen Mission ein, beschützte sie und schirmte sie ab. Die Illusion dieser ubiquitären Moderne ist leicht zu belegen, denn sie war nicht nur vom wirtschaftlichen Erfolg der Produzt:innen (und damit auch der Käufer:innen) dieses eher hochpreisigen Produkts abhängig, sondern auch vielfältig an Infrastrukturen gekettet, deren Überwindung eigentlich ihr Markenkern war.

## Danksagung

Dieser Aufsatz geht zurück auf meine Antrittsvorlesung als Professor für die Wissensgeschichte moderner Gesellschaften an der Helmut-Schmidt-Universität Hamburg im Oktober 2023. Ich möchte mich bei den Kolleg:innen von Fakultät und Fachgruppe für die konstruktiven Diskussionen, besonders aber bei den Mitgliedern der Professur für die äußerst produktive und unterstützende Zusammenarbeit bedanken. Darüber hinaus sei der Redaktion von NTM sowie zwei anonymen Gutachtenden für die hilfreichen Kommentare zu diesem Text gedankt.

## Footnotes

1 Über die Herkunft des Namens Jeep kursieren vielfältige Theorien. Am beliebtesten ist die angebliche Benennung nach der „Jeep“-Figur im bei den GIs populären Comic *Popeye*, jedoch trifft sie vermutlich nicht zu. Andere Versionen verweisen auf seinen „General Purpose“ oder eine rein technische Kennzahl. Zusammengetragen wurden diese vielfältigen Legenden unter anderem von der *Süddeutschen Zeitung*: „Rätsel auf Rädern“, *Süddeutsche Zeitung*, 08.09.2022.

2 Hagley Digital Archives, Taylor Vinson Collection of Transportation Ephemera, Series 1 Automobile Makes Box 1‑932, „Jeep Planning, 1945“.

3 1945 wurden durch die Reconstruction Finance Group folgende Preise festgelegt: Für einen Jeep der Baujahre 1943 bis 1945: 782 $, Baujahr 1942: 695 $, Baujahr 1941: 598 $; Hagley Digital Archives, National Automobile Dealers Association audiovisual materials and publications (Accession 2014.274), NADA2014001_NLTR_1945_12, Weekly News-Letter to Association Managers from NADA, No. 0202-0205, 07.12.1945.

4 Ebd.

5 Hagley Digital Archives, National Automobile Dealers Association audiovisual materials and publications (Accession 2014.274), NADA2014001_NLTR_1945_12, Weekly News-Letter to Association Managers from NADA, No. 0202-0205, 14.12.1945.

6 Die infrastrukturelle Durchdringung geht nach Tilley auch einher mit einem anderen Menschenbild der Kolonisierten, die in Bezug auf eine neue *development state*-Ideologie nicht mehr als politische Individuen, sondern als produktiv zu machende ökonomische Akteur:innen wahrgenommen werden.

7 „Le but poursuivi par les organisateurs est moins d’organiser une compétition sportive que d’attirer l’attention par une manifestation spectaculaire sur l’Afrique considérée comme une pièce importante des nouvelles politiques et stratégies mondiales et de s’acheminer ainsi vers une certaine communauté de vues africaines en préparant des liaisons plus intimes dans tous les domaines: économique, touristique, culturel,“ in: *Revue militaire d’information*, zitiert nach Boniface (2005: 63).

8 *Report Family Planning and Rural Health Services*, 28.09.1970; NARA College Park, Record Group 286, Entry P 970, Box 8.

9 UC Berkeley, Bancroft Library, Kaiser, (Henry J.) Papers, General Files 1944, Carton 24, folder 10.

10 Rollins paraphrasiert: „They [the SUVs] reflect characteristically ,postmodern‘ aspirations and anxieties, and in particular they embody a profoundly contradictory set of desires having to do with nature“ (S. 686).
